# Influence of altitude on cerebral and splanchnic oxygen saturation in critically ill children during air ambulance transport

**DOI:** 10.1371/journal.pone.0239272

**Published:** 2020-09-25

**Authors:** Tova Hannegård Hamrin, Staffan Eksborg, Jonas Berner, Urban Fläring, Peter J. Radell

**Affiliations:** 1 Pediatric Perioperative Medicine and Intensive Care, Astrid Lindgren Children's Hospital, Karolinska University Hospital Solna, Stockholm, Sweden; 2 Department of Physiology and Pharmacology, Section of Anesthesiology and Intensive Care, Karolinska Institutet, Astrid Lindgren Children's Hospital, Karolinska University Hospital Solna, Stockholm, Sweden; 3 Childhood Cancer Research Unit, Department of Women's and Children's Health, Karolinska Institutet, Astrid Lindgren Children's Hospital, Barnläkemedelsgruppen, Norrbacka S3:04, Karolinska University Hospital Solna, Stockholm, Sweden; Lundquist Institute at Harbor-UCLA Medical Center, UNITED STATES

## Abstract

**Objective:**

The aim of the current study was to investigate how cerebral and splanchnic oxygen saturation (rSO_2_-C and rSO_2_-A) in critically ill children transported in air ambulance was affected by flight with cabin pressurization corresponding to ≥ 5000 feet. A second aim was to investigate any differences between cyanotic and non-cyanotic children in relation to cerebral and splanchnic oxygen saturation during flight ≥ 5000 feet. The variability of the cerebral and splanchnic Near Infrared Spectroscopy (NIRS) sensors was evaluated.

**Design:**

NIRS was used to measure rSO_2_-C and rSO_2_-A during transport of critically ill children in air ambulance. rSO_2_ data was collected and stored by the NIRS monitor and extracted and analyzed off-line after the transport. Prior to evaluation of the NIRS signals all zero and floor-effect values were removed.

**Setting:**

The Pediatric Intensive Care Unit (PICU) at Astrid Lindgren Children’s Hospital, Karolinska University Hospital in Stockholm, Sweden.

**Patients:**

In total, 44 critically ill children scheduled for inter-hospital transport by a specialized pediatric transport team were included in the study between January 2014 and January 2019 (convenience sampling).

**Intervention:**

No interventions were conducted.

**Measurements:**

All study patients were monitored with a cerebral NIRS-sensor placed over the forehead and an abdominal NIRS-sensor placed in the infra-umbilical area for cerebral and splanchnic regional oxygen saturation monitoring, rSO_2_-C and rSO_2_-A, respectively.

**Main results:**

Complete rSO_2_-C and rSO_2_-A data was obtained in 39 patients. Median age was 12 days. Cyanotic congenital heart malformations were present in 9 patients (23%). In 22 patients (56%) rSO_2_-C decreased at altitude ≥ 5000 feet and in 24 patients (61%) rSO_2_-A decreased at altitude ≥ 5000 feet compared to baseline (p<0.0001). In 25 patients (64%) the rSO_2_-C/rSO_2_-A ratio was greater at altitude ≥ 5000 feet than at baseline. A ratio ≥ 1 was seen in 77% of patients at altitude ≥ 5000 feet compared to in 67% of patients at baseline.

**Conclusion:**

Both cerebral and splanchnic oxygen saturation decreased at altitude ≥ 5000 feet compared to baseline. In most patients, both cyanotic and non-cyanotic, cerebral oxygen saturation was preserved more than splanchnic oxygen saturation.

## Introduction

Specialized pediatric transport teams operate today as mobile intensive care units. They deliver advanced intensive care outside tertiary care centers for a wide variety of disorders using advanced monitoring equipment and skilled personnel [1–4]. Depending on distance between the referring and receiving hospital, some patients must be transported by air in helicopter or air-ambulance. Monitoring of oxygenation is important to ensure patient safety and the best possible patient outcome. Near-infrared spectroscopy (NIRS) is a noninvasive, method for monitoring of regional tissue oxygen saturation (rSO_2_) [5]. It has been shown that cerebral oxygen monitoring with NIRS detects changes in oxygenation earlier than pulse oximetry in periods of apnea during airway surgery in pediatric anesthesia [6].

NIRS-monitoring is attractive in neonatal and pediatric practice not only because it is noninvasive, but also because the penetration of the signal into the tissue corresponds well with the anatomy of neonates, infants and children [7]. Pediatric studies have demonstrated good correlation between cerebral rSO_2_ and jugular venous bulb saturation [8]. Anterior abdominal (splanchnic) rSO_2_ has shown strong correlation with gastric intra-mucosal pH as well as serum lactate and systemic venous oxygen saturation (SvO_2_) in children with congenital heart disease (CHD) [9]. In this group of patients with risk for low cardiac output, splanchnic rSO_2_ correlated better with systemic markers of oxygenation and perfusion such as serum lactate and SvO_2_ than did measurements over the renal bed. Multisite NIRS monitoring has been advocated to provide insight into the tissue response to different types of clinical interventions [10]. Somatic NIRS measurements may also be a better and earlier indicator of low cardiac output states than cerebral measurements, since the brain has efficient autoregulation [11].

During air transport the effects of altitude on patient oxygenation are of special importance. With increasing altitude, barometric pressure decreases and as a result also the partial pressure of oxygen. The result is lower oxygen saturations. Healthy children desaturated significantly, at times below 90%, in a hypoxic challenge test simulating the conditions of a commercial aircraft where the cabin is pressurized to 8000 feet, which equates to breathing 15% oxygen at sea level [12]. The effects of high altitude on critically ill children transported in air ambulance are not known. There are few studies of NIRS utilization in an air-transport environment and only two concerning pediatric patients [13, 14]. These studies were mainly performed in helicopters, with only 5 patients transported in air ambulance. The results suggested that cerebral oxygenation monitoring with NIRS can be used in a transport environment and that NIRS might be a useful complement to existing monitoring during inter-hospital transports.

In a previous methodological study, we found that the electronically stored NIRS data could be filtered and assessed off-line after the transport to improve reliability of the signal and thereby provide valuable post-hoc information about transport events [15].

The aim of the current study was to investigate how cerebral and splanchnic oxygen saturation in critically ill children transported in air ambulance was affected by flight ≥ 5000 feet. A second aim was to investigate any differences between cyanotic and non-cyanotic children in relation to cerebral and splanchnic oxygen saturation. Finally, we evaluated the variability of both cerebral and splanchnic NIRS sensors.

## Material and methods

### Ethical approval

Ethical approval for this study was provided by the Regional Ethics Review Board of Stockholm, Sweden (DNr 2013/1487-31/1 and 2016/2036-32).

### Study design and population

This was a prospective observational study, registered in the Australian New Zealand Clinical Trials Registry (ANZCTR) with registration number ACTRN12619001710112. Following written parental informed consent, 44 critically ill children scheduled for inter-hospital transport by a specialized pediatric transport team at the Pediatric Intensive Care Unit (PICU) at Astrid Lindgren Children’s Hospital, Karolinska University Hospital in Stockholm, were enrolled in the study between January 2014 and January 2019 (convenience sampling). Exclusion criteria were lack of consent, participation in any other clinical research study, flights with an estimated duration less than 50 minutes and flights with a need for sea level cabin altitude. Transports were both acute and planned transfers to and from the PICU at Astrid Lindgren Children’s Hospital. The team is staffed by a PICU consultant and a specialist anesthesia or intensive care registered nurse with a minimum of 3 years experience in pediatric anesthesia or pediatric intensive care [3].

### Equipment and procedures

Patients were transported in Beech Superking Air 200 and Cessna Citation II 550 air ambulances.

Standard monitoring during transport, including pulse oximetry, electrocardiographic monitoring, blood pressure measurements, body temperature, respiratory rate and evaluation with Comfort-B scale for level of sedation/comfort, was performed in all study patients and checked and noted in the study protocol before transport (base-line), during flight with cabin pressurization corresponding to ≥ 5000 feet and after transport [16]. The transport risk index of physiological stability (TRIPS) score was recorded before, during transport at ≥ 5000 feet and after transport for neonatal patients ≤ 30 days at the time of transport [17].

All study patients were monitored with a cerebral NIRS-sensor placed over the forehead and an abdominal NIRS-sensor placed in the infra-umbilical area for cerebral and splanchnic regional oxygen saturation monitoring, rSO_2_-C and rSO_2_-A respectively (INVOS-5100C, Covidien, Mansfield, MA, USA). The sensors had the following dimensions: 17.25 cm^2^ for neonates and infants and 28.8 cm^2^ for pediatric patients. The probes had two light paths with an emitter/diode spacing of 30–40 mm and a light penetrating depth of 20–40 mm. Monitoring began at the hospital before patient transport and was continued during transfer in ground ambulance to and from the airport as well as during air ambulance transport and was finished upon arrival at the receiving hospital. Cerebral and splanchnic rSO_2_ data were stored by the INVOS monitor during transport and extracted and analyzed off-line after the transport. The data points had a spacing of 6 seconds.

Transport personnel were instructed not to make clinical care decisions based on values presented on the INVOS monitor. Therapeutic interventions, including adjustment of fraction of inspired oxygen (FiO_2_), were made according to the clinical judgement of the transport team. To reduce ambient light exposure, aluminum foil was used to cover the cerebral probe and the abdominal probe was covered under the patient´s clothes and blankets. The internal battery time of the INVOS 5100C is approximately 20 minutes, which made access to an external power supply necessary for both ground and air ambulance. The NIRS data was downloaded from the monitor using a Microsoft Excel 2010 spreadsheet (Microsoft Corp., Washington, DC, USA).

Prior to evaluation of the NIRS signals all zero and floor-effect values were removed. For visual inspection of NIRS signals the Savitzky–Golay algorithm of smoothing and differentiation of data by simplified least square procedures (least-squares fitting using 20 and 50 neighbors; 2nd order polynom) was applied to perform noise reduction in the signal [15, 18].

To further evaluate differences between cerebral and splanchnic oxygenation, the cerebral—splanchnic ratio (rSO_2_-C/rSO_2_-A) for each patient at baseline and at altitude ≥ 5000 feet was calculated.

### Statistics

Data is presented as median and inter-quartile range. All statistical evaluation was performed on non-smoothed data. The variability for rSO_2_-values were expressed by the coefficient of variation and compared with the Friedman’s test with the Dunn’s multiple comparison test. Wilcoxon signed-rank test was used for the comparison of column medians to a hypothetical value. Several independent populations were compared with Kruskal-Wallis statistics with the Dunn’s multiple comparison test.

Statistics were evaluated by MS Excel (Microsoft Corporation, Redmond, Washington, USA) and Graph Pad Prism version 5.04 (Graph Pad Software Inc. San Diego, USA). All statistical tests were two sided and p values < 0.05 were considered to be statistically significant.

## Results

**Fig 1:**
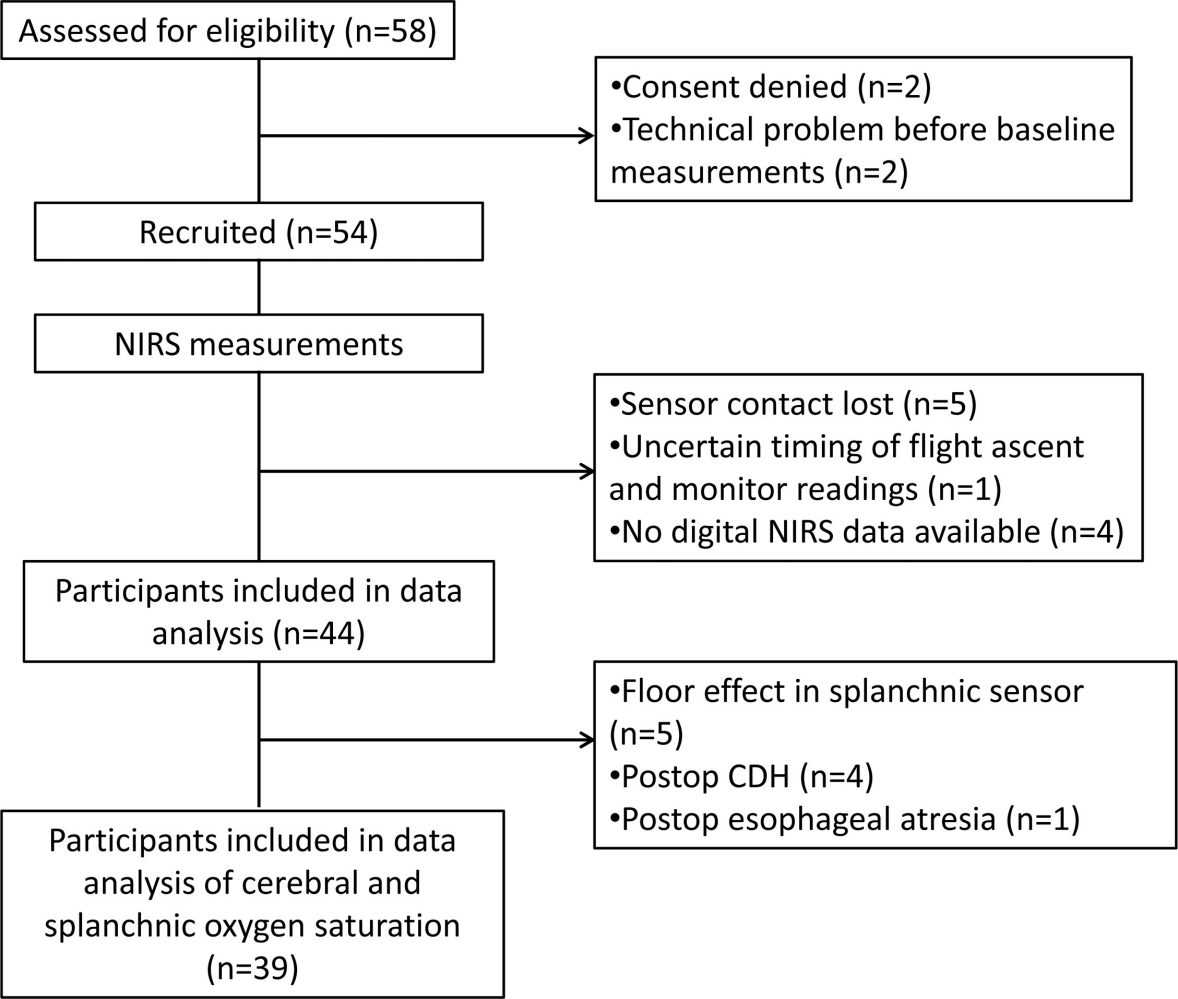
Recruitment process of study participants. NIRS = Near-infrared Spectroscopy. CDH = Congenital Diaphragmatic Hernia.

In total 44 patients were monitored. Complete cerebral regional oxygen saturation (rSO_2_-C) and splanchnic regional oxygen saturation (rSO_2_-A) data were obtained in 39 patients (Fig 1).

Median age was 12 days and median weight 3.55 kg. Cyanotic congenital heart malformations were present in 9 patients (23%). Mechanical ventilation was used in 12 patients (31%), 4 patients (10%) needed CPAP and 4 patients (10%) needed support with high flow nasal cannula (Table 1). Patients were categorized as cyanotic due to the presence of an intracardiac lesion or ductus arteriosus with a significant right to left shunt/mixing affecting the systemic saturation as measured by the SpO_2_. Diagnoses that were grouped into cyanotic congenital heart malformations were: Transposition of the Great Arteries (TGA) n = 3; Pulmonary Atresia n = 2; Pulmonary Atresia & Tricuspid Atresia n = 2; Total Anomalous Pulmonary Venous Return (TAPVR) n = 1 and Truncus Arteriosis n = 1. Median pulse oximetry registrations at Take-Off were 89% (IQR 83.5–93) and 99% (IQR 96–100) for cyanotic patients and non-cyanotic patients, respectively (p<0.0001).

**Table 1:** Patient characteristics.

Patient No.	Age (days)	Weight (kg)	Sex	Diagnosis	Cabin altitude (feet)	Barometric pressure (kPa)	Breathing support	FiO_2_ start	Change FiO_2_ at altitude	PiO_2_ (kPa)	SpO_2_% min	SpO_2_% max	Hb (g/L)	Transport time (min)
1	1	3.7	M	Interrupted aortic arch, ASD + VSD	6000	81.22	sp	0.21	No	15.7	87	94	175	70
2	12	3.3	M	CoA + VSD	6000	81.22	CPAP	0.21	No	15.7	99	100	148	70
3*	1	3.8	F	PA with intact ventricular septum	6000	81.22	sp	0.21	No	15.7	83	87	149	70
4	5	5.1	M	PPHN, biventricular failure	5000	84.33	sp	0.21	No	16.4	85	98	182	75
5	109	4.8	M	Dystrofia Myotonica type I	6000	81.22	BiPaP	0.21	1 litre O_2_	18.0	85	100	₋	50
6*	9	4.1	F	PA and TA, Post op status after BT-shunt	6000	81.22	sp	0.21	No	15.7	68	88	₋	72
7*	8	3.8	M	TGA, septostomy	6000	81.22	sp	0.21	No	15.7	75	82	151	75
8	24	3.7	M	Aortic Stenosis	7400	77.00	sp	0.21	No	14.8	97	100	150	81
9	23	3.5	M	Post op status after CDH	6900	78.49	HFNC	0.3	0.5	36.1	75	100	109	70
10*	2	3.2	M	PA and TA	6000	81.22	intubated	0.21	No	15.7	84	94	173	75
11	1136	13.0	F	Hemolytic uremic syndrome, anuria	5800	81.84	sp	0.21	No	15.9	95	100	86	78
12	4	3.8	F	Tracheomalacia	6000	81.22	sp	0.21	No	15.7	85	96	₋	99
13	8	3.2	F	Sepsis, status after ECMO	6000	81.22	intubated	0.38	No	28.5	98	100	₋	199
14	12	4.1	M	MAS, status after ECMO	6000	81.22	intubated	0.46	No	34.5	90	94	142	60
15	33	1.4	M	Aortic Stenosis, Left chamber hypertrophy	6000	81.22	CPAP	0.55	1	74.9	90	95	₋	68
16	3	3.4	M	CoA	6000	81.22	sp	0.21	No	15.7	86	99	₋	71
17	39	1.4	M	Post op status after commissurotomy of aorta + PDA ligation	6000	81.22	intubated	0.45	No	33.7	93	100	109	76
18*	24	3.2	M	PA, Post op status after BT-shunt	6000	81.22	sp	0.21	No	15.7	77	86	₋	75
19	7	3.6	F	CoA + VSD	6000	81.22	sp	0.21	No	15.7	93	100	207	82
20	2	3.7	M	CoA + VSD	6000	81.22	sp	0.21	1 litre O_2_	18.0	83	100	179	80
21	10	3.0	M	MAS, status after ECMO	6000	81.22	HFNC	0.3	No	22.5	90	100	109	70
22	14	3.9	M	CoA + VSD	5120	83.96	sp	0.21	No	16.3	92	98	151	82
23*	5	2.2	M	TAPVR + ASD	6000	81.22	intubated	0.5	No	37.5	85	89	₋	66
24	58	3.4	M	Post op status after CDH, status after ECMO	6000	81.22	intubated	0.4	0.5	37.5	86	100	124	207
25	3	2.5	M	Interrupted aortic arch, ASD + VSD	6000	81.22	sp	0.21	No	15.7	98	100	172	71
26	12	4.3	F	MAS, status after ECMO	6000	81.22	sp	0.21	3 litre O_2_	24.0	79	100	105	75
27*	4	3.9	M	TGA, septostomy	6000	81.22	sp	0.21	No	15.7	85	94	170	72
28	5	3.9	F	PPHN, asphyxia. Status after ECMO	6000	81.22	intubated	0.35	No	26.1	96	100	122	181
29*	3	3.4	M	TGA, septostomy	6000	81.22	intubated	0.21	No	15.7	84	93	142	67
30	2	3.4	M	CoA	6000	81.22	sp	0.21	No	15.7	95	100	195	87
31	59	3.4	F	ASD + VSD with heart failure	5900	81.53	intubated	0.25	No	18.8	96	100	112	60
32	1148	15.0	F	Neck abscess	6000	81.22	sp	0.21	No	15.7	95	100	97	63
33	9	4.4	M	MAS + sepsis, status after ECMO	6000	81.22	intubated	0.45	No	33.7	95	100	113	211
34	160	6.6	M	BPD, ex premature	7000	78.19	intubated	0.3	0.4	28.8	86	100	127	75
35	87	2.4	M	Ex premature. Post op PDA ligation	6000	81.22	HFNC	0.3	0.35	26.2	89	100	₋	142
36*	3	3.2	F	Truncus arteriosis	6000	81.22	CPAP	0.21	No	15.7	86	95	196	84
37	701	6.9	M	Post op status after AVSD. PPHN.	6000	81.22	HFNC	0.7	No	52.4	96	100	98	72
38	162	6.3	F	Tracheal stenosis	6000	81.22	sp	0.21	No	15.7	93	100	₋	148
39	19	3.5	M	Post op status after CDH, status after ECMO	6000	81.22	intubated	0.3	0.34	25.5	89	100	127	156

FiO_2_ = fraction of inspired oxygen, SpO_2_ = peripheral capillary oxygen saturation, ECMO = Extracorporeal membrane oxygenation, CoA = Coarctation of the aorta, PPHN = Persistent Pulmonary Hypertension in the Newborn, MAS = Meconium Aspiration Syndrome, BPD = Bronchopulmonary dysplasia, CHD = Congenital Heart Disease, CDH = Congenital Diaphragmatic Hernia, TGA = Transposition of the Great Arteries, PDA = Patent Ductus Arteriosus, TAPVR = Total Anomalous Pulmonary Venous Return, BT-shunt = Blalock-Taussig shunt, ASD = Atrial Septal Defect, VSD = Ventricular Septal Defect, sp = Spontaneous breathing, CPAP = Continuous Positive Airway Pressure, BiPaP = Biphasic Positive Airway Pressure, HFNC = High-Flow Nasal Cannula, PA = Pulmonary Atresia, TA = Tricuspid Atresia

* = cyanotic CHD. PiO_2_ given is with FiO_2_ added/increased where applicable.

Simultaneous rSO_2_-C and rSO_2_-A values for each patient were investigated before flight (departure from hospital ward—loading into ambulance–transport in ambulance—take-off), at altitude ≥ 5000 feet and after flight (landing–unloading–transport in ambulance—arrival at receiving hospital) after all zero values and floor effect values had been removed. Simultaneously registered numbers of readings for rSO_2_-C and rSO_2_-A before flight were 708 (IQR 521–1140), median registration time 53 minutes (IQR 43–71). At altitude ≥ 5000 feet the number of values simultaneously registered was 255 (IQR 132–435), median registration time 15 minutes (IQR 29–43) and after flight 521 (IQR 358–607), median registration time 53 minutes (IQR 41–61).

**Fig 2:**
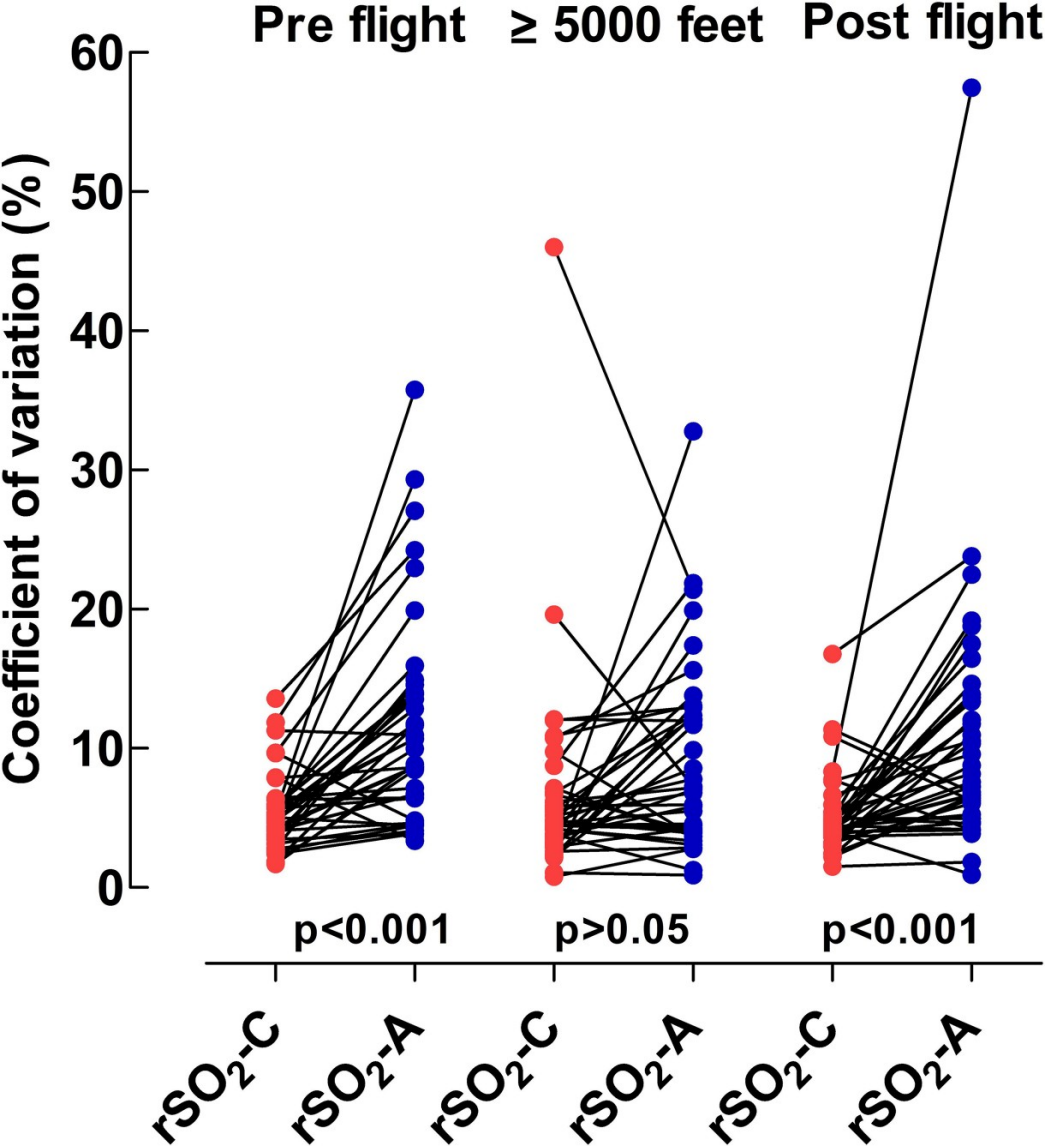
Coefficient of variation demonstrating the scatter for rSO_2_-C and rSO_2_-A in each patient before flight, at altitude ≥ 5000 feet and after flight. All recorded values after all zero values and floor effect values were removed are used. Red dots = rSO_2_-C, Blue dots = rSO_2_-A.

No statistically significant difference in variability was seen within rSO_2_-C or within rSO_2_-A when values before flight were compared to values at altitude ≥ 5000 feet and after flight. Overall, there was greater variability seen in rSO_2_-A measurements than in rSO_2_-C (p<0.0001), Fig 2.

Changes in rSO_2_-C and in rSO_2_-A between pre-flight, flight at altitude ≥ 5000 feet and after flight were investigated for each patient using all recorded values. There was a statistically significant difference (p<0.0001) and post-hoc tests for each patient and each sensor were performed (Table 2).

**Table 2:** Regional oxygen saturation values for all patients.

	rSO_2_-C (%)						rSO_2_-A (%)					
Patient No.	Pre flight		≥5000 feet		Post flight		Pre flight		≥5000 feet		Post flight	
1	81	(80–82)	79	(77–80)	85	(84–86)	50	(45–56)	43	(41–46)	67	(65–70)
2	71	(68–74)	72	(68–74)	72	(71–76)	50	(47–53)	61	(56–63)	57	(52–60)
3*	61	(60–63)	64	(62–65)	62	(61–63)	55	(49–60)	48	(44–52)	55	(46–59)
4	79	(75–82)	70	(67–72)	78	(76–81)	78	(74–83)	66	(65–67)	78	(76–81)
5	64	(63–66)	75	(66–78)	70	(67–73)	66	(60–71)	35	(31–42)	70	(66–73)
6*	63	(61–65)	61	(58–62)	65	(62–67)	54	(51–59)	48	(42–49)	48	(44–56)
7*	61	(59–64)	57	(55–59)	62	(60–63)	48	(46–49)	51	(48–56)	49	(45–52)
8	80	(77–81)	78	(75–79)	80	(78–82)	86	(78–91)	83	(80–85)	81	(76–84)
9	63	(60–67)	61	(60–62)	75	(73–76)	44	(36–51)	37	(33–39)	52	(47–58)
10*	76	(61–78)	66	(62–69)	71	(69–74)	43	(37–49)	46	(44–48)	41	(31–46)
11	68	(67–70)	62	(60–63)	67	(66–68)	58	(51–64)	45	(42–51)	62	(58–68)
12	71	(69–72)	58	(57–61)	67	(66–69)	93	(90–94)	76	(72–78)	88	(85–89)
13	64	(62–65)	65	(64–66)	69	(66–70)	74	(72–77)	47	(43–51)	49	(44–55)
14	86	(85–88)	90	(89–90)	86	(85–88)	61	(57–64)	49	(48–50)	59	(56–61)
15	82	(76–84)	73	(71–76)	80	(75–84)	70	(65–73)	72	(69–74)	71	(65–74)
16	80	(76–83)	71	(69–73)	79	(77–82)	91	(88–93)	75	(74–77)	86	(84–88)
17	82	(79–86)	69	(67–70)	72	(71–74)	37	(28–50)	19	(15–23)	33	(28–37)
18*	51	(50–52)	40	(38–43)	54	(51–56)	44	(42–46)	32	(29–36)	52	(49–54)
19	82	(79–87)	77	(76–78)	80	(77–83)	83	(79–87)	69	(66–73)	69	(65–72)
20	86	(84–88)	80	(77–84)	87	(86–89)	69	(67–70)	61	(59–65)	73	(71–75)
21	66	(63–68)	63	(62–65)	66	(64–68)	68	(59–72)	64	(62–65)	67	(64–69)
22	71	(68–73)	64	(62–66)	77	(75–79)	78	(72–83)	59	(57–61)	62	(58–65)
23*	64	(62–66)	63	(63–63)	67	(66–67)	66	(62–69)	64	(64–64)	63	(62–64)
24	64	(62–66)	69	(68–70)	72	(71–73)	54	(50–57)	40	(34–47)	44	(37–48)
25	62	(58–68)	76	(73–80)	70	(66–73)	66	(64–68)	67	(63–68)	69	(67–71)
26	64	(63–66)	59	(57–71)	66	(65–68)	73	(67–78)	53	(49–60)	59	(55–63)
27*	63	(61–66)	52	(50–53)	63	(62–65)	54	(51–57)	49	(41–53)	47	(43–52)
28	78	(75–81)	70	(50–74)	79	(77–80)	58	(51–64)	69	(67–74)	74	(70–75)
29*	74	(69–81)	67	(65–69)	72	(70–75)	57	(49–66)	64	(62–65)	70	(68–72)
30	76	(73–81)	69	(64–72)	73	(69–77)	86	(84–88)	78	(76–80)	49	(18–85)
31	74	(71–76)	73	(71–74)	70	(68–72)	59	(55–64)	72	(67–72)	72	(69–75)
32	66	(65–67)	55	(53–57)	64	(63–65)	70	(68–72)	56	(54–58)	73	(71–75)
33	71	(69–73)	67	(64–69)	67	(66–68)	62	(45–73)	62	(56–66)	73	(60–76)
34	80	(77–81)	80	(76–81)	82	(81–87)	72	(71–74)	74	(73–74)	76	(76–77)
35	60	(55–65)	62	(57–65)	55	(45–57)	61	(57–73)	66	(59–71)	55	(45–68)
36*	67	(65–68)	68	(67–69)	66	(64–67)	68	(65–72)	71	(69–72)	67	(64–69)
37	65	(63–67)	57	(55–58)	55	(52–59)	47	(42–50)	53	(50–55)	48	(46–50)
38	89	(87–91)	91	(87–92)	90	(88–92)	72	(70–74)	70	(67–72)	72	(70–74)
39	73	(72–76)	76	(66–79)	75	(73–77)	51	(48–57)	55	(45–61)	40	(37–43)
Cyanotic	63	(61–71)	63	(55–67)	65	(62–69)	54	(46–62)	49	(47–64)	52	(48–65)
NON-cyanotic	72	(65–80)	70	(63–76)	73	(67–80)	67	(57–75)	62	(49–71)	68	(54–73)

rSO_2_-C = cerebral oxygen saturation. rSO_2_-A = splanchnic oxygen saturation. Data are expressed as median values (IQR).

* = cyanotic CHD.

The data contained in Table 2 are summarized at the bottom of the table as median (IQR) for patients with and without cyanotic heart disease.

The relationship between rSO_2_-C, rSO_2_-A and the TRIPS score was investigated for 28 neonatal patients. Either rSO_2_-C or rSO_2_-A were affected in the patients (n = 10) where the TRIPS score increased between pre-transport and flight at ≥ 5000 feet (p = 0.30 and p = 0.2, respectively). Patients were grouped into three different categories with increasing pre-transport TRIPS scores 0–10, 11–20 and 21–30. The distribution of TRIPS score categories at different times is shown in Table 3. There was a decrease in TRIPS score after transport resulting in fewer patients in TRIPS category 21–30 compared to pre-transport. In most patients, the TRIPS score did not change during transport.

**Table 3:** TRIPS score.

TRIPS score category	Pre-transport (n)	Take-Off (n)	Altitude ≥5000 feet (n)	Post flight (n)	At receiving hospital (n)
**0–10**	**20**	**19**	**20**	**20**	**20**
**11–20**	**2**	**3**	**5**	**5**	**5**
**21–30**	**6**	**6**	**3**	**3**	**3**

Pre-transport TRIPS and change in TRIPS scores during and after transport. TRIPS = The transport risk index of physiological stability. n = number.

**Fig 3:**
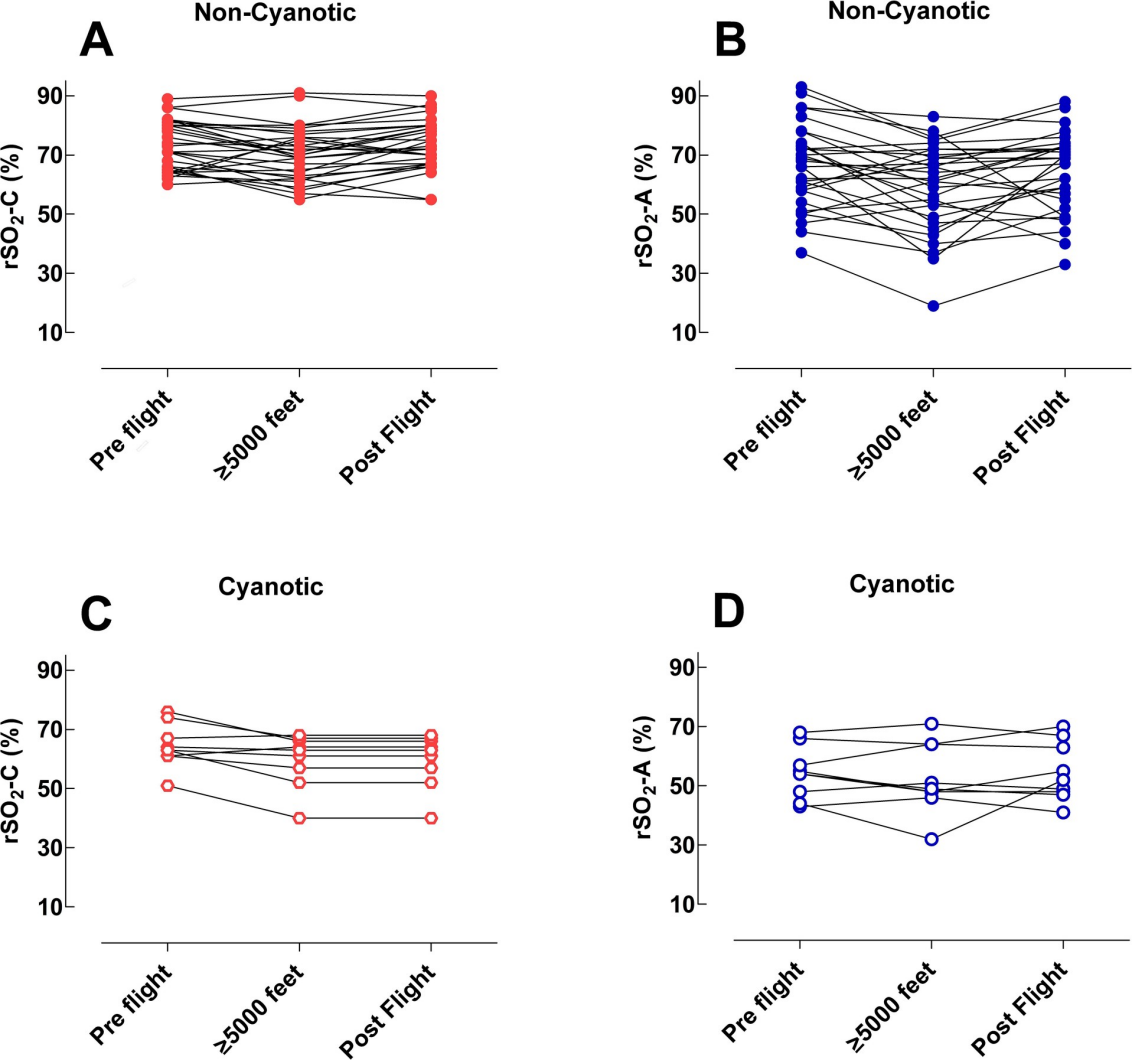
The median values of rSO_2_-C and rSO_2_-A. Data were derived from all recorded values, determined for rSO_2_-C and rSO_2_-A in each individual patient at baseline, at altitude ≥ 5000 feet and after flight. 3A: rSO_2_-C in non-cyanotic patients, 3B: rSO_2_-A in non-cyanotic patients, 3C: rSO_2_-C in cyanotic patients and 3D: rSO_2_-A in cyanotic patients.

Older patients (n = 10) were monitored with Comfort-B scores for level of sedation, there was no statistically significant difference in the Comfort B levels between pre-flight, flight at altitude ≥ 5000 feet and after flight. In 22 patients (56%) rSO_2_-C decreased at altitude ≥ 5000 feet compared to baseline, in 9 patients there was no significant difference and in 8 patients rSO_2_-C increased at altitude. In 24 patients (61%) rSO_2_-A decreased at altitude ≥ 5000 feet compared to baseline, in 8 patients there was no statistically significant difference and in 7 patients rSO_2_-A increased at altitude. In patients with cyanotic heart malformations the rSO_2_-C value at altitude ≥ 5000 feet decreased compared to baseline in 6 of 9 patients and rSO_2_-A decreased in 4 patients. These findings are illustrated by plotting of the median values for rSO_2_-C and rSO_2_-A in each patient at baseline, altitude ≥ 5000 feet and after flight. Cyanotic and non-cyanotic patients are presented separately (Fig 3A–3D).

The quotients rSO_2_-C/rSO_2_-A were > 1 in 26 patients (67%) at baseline and in 30 patients (77%) at altitude ≥ 5000 feet. The ratio shifted from being < 1 at baseline to being > 1 at altitude in seven patients and in four patients the ratio shifted from being > 1 at baseline to being < 1 at altitude. In 25 patients (64%) the rSO_2_-C/rSO_2_-A ratio was greater at altitude ≥ 5000 feet than at baseline. A statistically significant difference between rSO_2_-C/rSO_2_-A at baseline and at altitude was found in 36 subjects (92.3% of patients).

**Fig 4:**
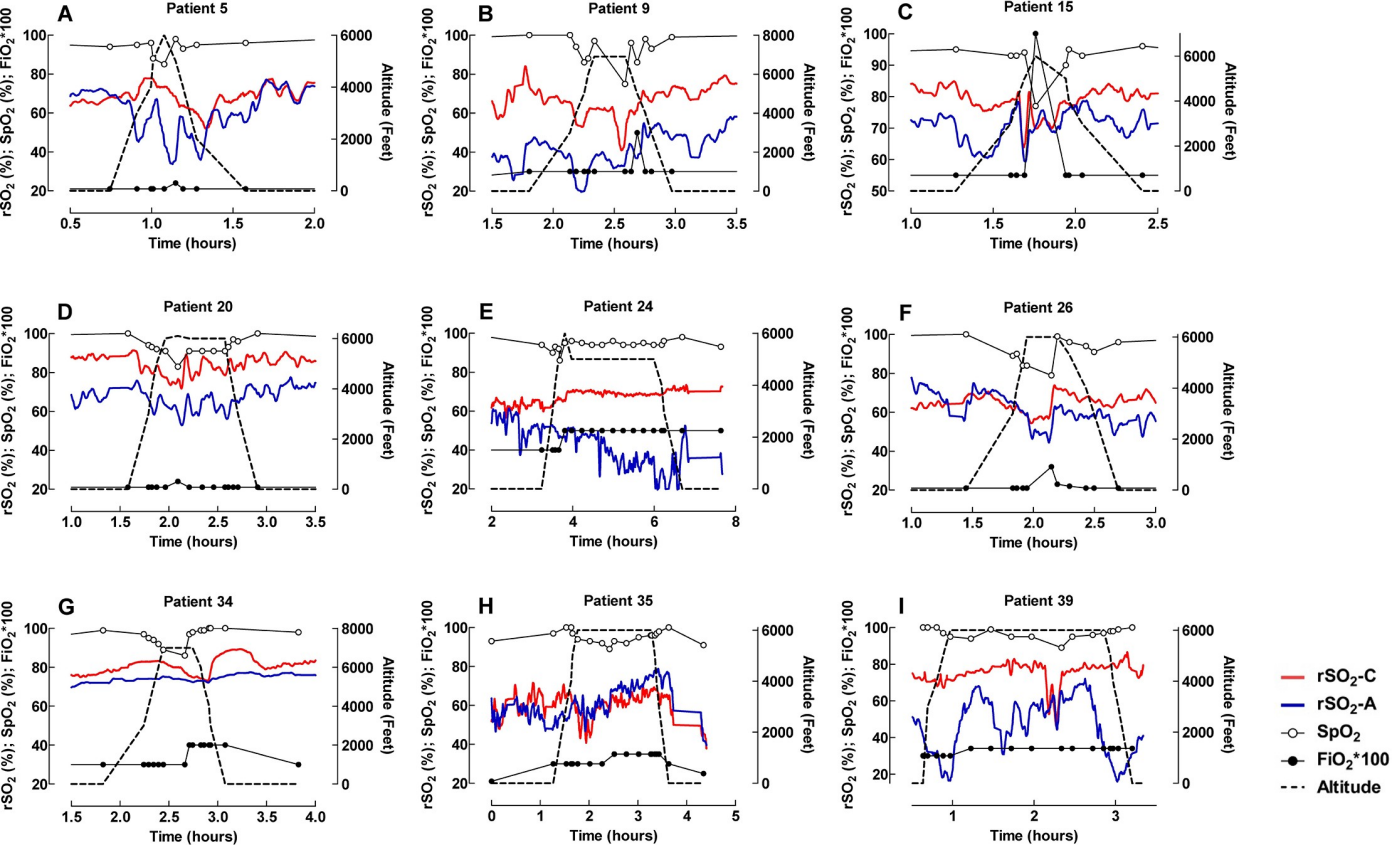
Changes in SpO_2_, rSO_2_-C and rSO_2_-A with an increase in FiO_2_ illustrated for nine patients who required additional oxygen during flight. The influence of altitude on rSO_2_-C, rSO_2_-A and pulse oximetry for 3 different patients was visualized after smoothing with 20 neighbours. The graphs reveal different characteristic patterns of reacting to altitude (Fig 5A–5C).

Nine patients required additional oxygen during flight. The differences and responses seen in SpO_2_, rSO_2_-C and rSO_2_-A with an increase in FiO_2_ are illustrated in Fig 4 for all nine patients. In five patients: two spontaneously breathing, one on BiPaP, one on CPAP and one on HFNC, oxygen supply was changed (added or increased) during a limited time (minute to 15 minutes) of the flight (Fig 4A–4E). In the remaining four patients: three intubated patients and one patient on HFNC, oxygen was increased and continued at the higher FiO_2_ level during the remaining part of the transport (Fig 4F–4I).

**Fig 5:**
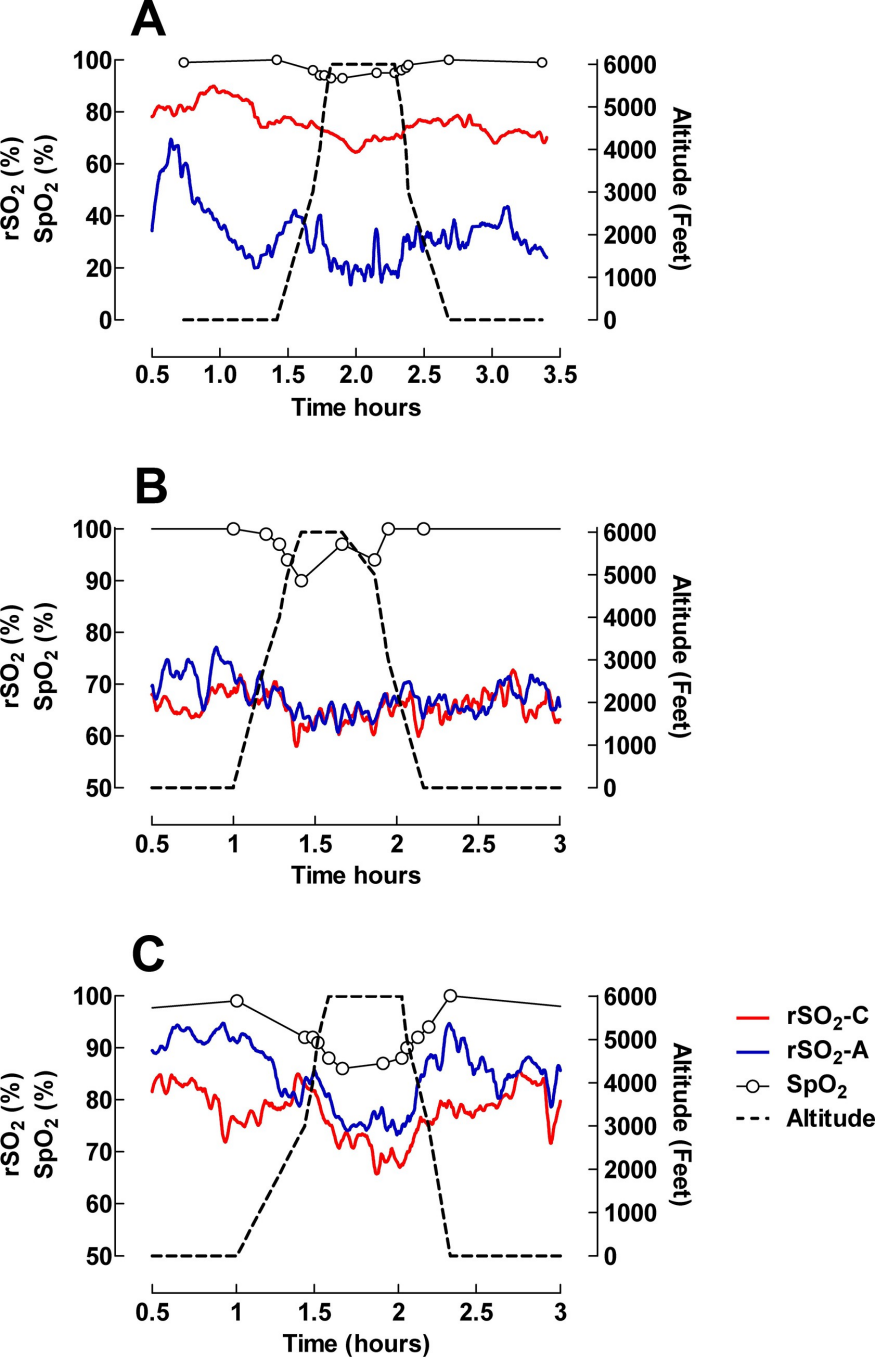
Different characteristic patterns of reacting to altitude in 3 different patients. rSO_2_-C was higher, at the same level and lower than rSO_2_-A in Fig 5A (Patient 17), 5B (Patient 21) and 5C (Patient 16), respectively. The NIRS curves were smoothed by the Savitzky–Golay filtering method (20 neighbours) [16].

## Discussion

To the best of our knowledge this is the first study to investigate regional tissue oxygen saturation with multisite registrations from both cerebral and splanchnic areas during inter-hospital transport of critically ill children. It is also the first study which has focused on monitoring of regional tissue oxygen saturation during inter-hospital transportation of critically ill children in air ambulances.

To evaluate the consistency in measurements within each sensor throughout the entire transport event for every patient as well as between rSO_2_-C and rSO_2_-A sensors we studied the coefficient of variability (Fig 2). We found that the transport process per se had no influence on the variability in either sensor. Our findings of a greater variability in rSO_2_-A when compared to rSO_2_-C are consistent with others [19]. All patients, including those mechanically ventilated, were transported without using muscle relaxants as this is our clinical routine. The greater variability seen in rSO_2_-A could result from the abdomen being a more mobile body area compared to the forehead, also in small children. Interestingly, we found that all patients excluded due to poor signal acquisition in the splanchnic sensor, had had previous abdominal surgery (Fig 1).

This study showed that in a majority of patients rSO_2_-C and rSO_2_-A had a statistically significant decrease at altitude ≥ 5000 feet compared to baseline. (Fig 3, Table 2). A large amount of data has been collected in this study and values should be interpreted and valued in a clinical perspective, since also small differences show statistical significance. Previous research has defined low rSO_2_-C as a continuous decrease of > 20% from baseline [20]. When analyzing our data in this perspective we found a decrease of 20% in median values in only one patient. This patient (Patient 18), who belonged to the cyanotic group of patients, showed a simultaneous decrease in SpO_2_. rSO_2_-A values in healthy newborns were found to resemble rSO_2_-C values by 48 h postnatal age [20]. We found 4 patients who had a 20% reduction from baseline in rSO_2_-A, one without a simultaneous decrease in SpO_2_. No clinical deterioration was observed in this patient during the transport, and therefore, no clinical interventions occurred. By evaluating changes in rSO_2_-C and rSO_2_-A in relation to SpO_2_, we found that 6 patients showed a substantial decrease in SpO_2_, without a corresponding decrease in rSO_2_-C and rSO_2_-A, interestingly 3 of these patients belonged to the cyanotic group. A more profound decrease in rSO_2_-C and/or rSO_2_-A than in SpO_2_ was found in 5 patients, most obvious in rSO_2_-A.

By using the rSO_2_-C/rSO_2_-A ratio we wanted to compare regional oxygen saturation of cerebral and splanchnic tissue in this cohort and investigate if a relative change could be detected at ≥ 5000 feet as an effect of altitude. A ratio ≥ 1 was seen in a majority of patients at baseline and in even more patients (77%) at altitude ≥ 5000 feet (FiO_2_ was increased in only 2 patients). Among patients with a ratio < 1 at baseline, all but 2 had an elevated ratio closer to 1 at altitude. We speculate that an increasing ratio might imply that cerebral tissue was protected by auto-regulation in this increasingly hypoxic environment. We found no association between age, breathing support or diagnosis in patients with a ratio < 1. Ideally oxygen extraction would have been a more reliable measurement which could have provided additional information, but we were not able to extract continuous data from the pulse oximetry device, which limited this possibility.

We were concerned about cyanotic patients and the effects of altitude on rSO_2_, but we found no specific patterns in rSO_2_-C or rSO_2_-A in relation to altitude in this group of patients, regardless of breathing support. We did note however that 7 of 9 (78%) cyanotic patients had a rSO_2_-A value < 60% at baseline but only 10 of 30 (30%) non-cyanotic patients, and that all but one patient in the cyanotic group (Total Anomalous Pulmonary Venous Return) showed a decrease in SpO_2_ at altitude ≥ 5000 feet.

We found no clear relationship between certain diagnosis and the effect of altitude. Our cohort had several patients with congenital heart disease, which could possible complicate assessment, but different reactions were also seen among other patients. This is illustrated in Fig 4 where 4A shows a spontaneously breathing patient with TGA on Prostaglandin E1 infusion and after balloon atrial septostomy, 4B a patient with high flow nasal cannula after ECMO treatment for meconium aspiration and 4C a spontaneously breathing patient with coarctation of the aorta without a patent ductus arteriosus.

Caution should be paid to meticulous application and protection of sensors in order to reduce the number of artefacts, and values should be interpreted in relation to clinical assessments. We found that measurements of rSO_2_-A involved more difficulties than rSO_2_-C, such as greater variability and a higher number of artefacts, but also gave more information in addition to measurements from pulse oximetry than did rSO_2_-C.

Limits of this study were that SpO_2_ values from pulse oximetry were single values and not from continuous measurements which made calculations of fractional tissue oxygen extraction unreliable. The difficulties in obtaining reliable measurements on oxygen extraction is explained in more detail in supplementary materials. Infants with cyanotic congenital heart disease may have significant preductal/post-ductal SpO_2_ differences and the lack of both pre -and post-ductal registrations with SpO_2_ during the flight protocol is a weakness. Therefore, the potential for differences in arterial saturation across the aortic arch confounds interpretation of regional rSO_2_ changes in these patients. Changes to NIRS readings not solely based on changes in altitude is a possible source of confounding. These changes could be due to clinical severity. A transport risk index of physiologic stability (TRIPS) score was therefore used to further group neonatal patients based on their clinical severity and the effect on rSO_2_ during the transport. In most patients, the TRIPS score did not change during transport. Furthermore, we found no evidence that changes in rSO_2_-readings were due to other clinical severity, as rSO_2_-readings were not affected in patients who had a higher TRIPS score at altitude ≥ 5000 feet than pre-transport.

In contrast the strengths of the current study were the large number of NIRS measurements collected both at baseline and during transport at altitude and our ability to remove nonsense values such as zero values and floor effect values prior to evaluation to limit the impact of artefacts on the results.

An important question on transport, where equipment needs to be limited due to space and weight, is what NIRS would add to the existing monitoring system and clinical management, and ultimately if patients’ outcome would be better if NIRS is added compared to simply monitoring with pulse oximetry. Our opinion is that NIRS should be added, during transport, to those patients where the clinical management during anesthesia and intensive care would have included monitoring with NIRS in a hospital setting. Namely situations where measurements of venous saturations provide a means of titrating medications such as inotropes, vasoactive medicines and volume, or other situations with very low perfusion and oxygenation conditions in which traditional pulse oximetry could fail. To other patients, NIRS monitoring during transport is probably superfluous and would not add any clinical utility.

## Conclusions

Both cerebral and splanchnic oxygen saturation decreased at altitude ≥ 5000 feet compared to baseline in a majority of patients. In most patients cerebral oxygen saturation was preserved more than splanchnic oxygen saturation. This was also the case for cyanotic patients even though low baseline splanchnic oxygen saturation values were observed in most cyanotic patients. The transport process per se had no influence on the variability in either sensor. NIRS-monitoring may be useful in the transport environment in the same clinical situations where it would have been used in a hospital setting. To other patients, NIRS monitoring during transport is probably superfluous and would not add any clinical utility to existing monitoring. Future studies should include equivalent continuous data for pulse oximetry for calculation of oxygen extraction to further determine the usefulness of regional oxygen saturation monitoring during transport.

## Supporting information

S1 File (DOCX)
