# Supplementary material for: Influence of altitude on cerebral and splanchnic oxygen saturation in critically ill children during air ambulance transport
Source: PLoS One. 2020 Sep 25;15(9):e0239272. doi: 10.1371/journal.pone.0239272 (PMC7518599; doi:10.1371/journal.pone.0239272)
Supplement: S1 File — (DOCX) [file pone.0239272.s001.docx]

**Precision of fractional tissue oxygen extraction**

To evaluate the precision of estimated fractional tissue oxygen extraction the mean and standard deviation (SD) for rSO_2_-C and rSO_2_-A respectively was calculated, using values for 2 minutes, starting 1 minute before the time of the SpO_2_ measurement. SD for SpO_2_ was assumed to be 0 since the value given on the pulse oximetry monitor is an average value and there was very little variability during the entire registered period. The coefficient of variation (100*SD/FTOE) was calculated including propagation of errors involving subtraction and division according to JE Parks <http://www.phys.utk.edu/labs/plproerr.pdf>.

The supplementary figures showed a statistically significant increase in coefficient of variation (CV) with decreasing fractional tissue oxygen extraction. The CV was of such a magnitude that calculations on fractional tissue oxygen extraction bear no interest and there is a risk that erroneous conclusions could be drawn.
