# Supplementary material for: Influence of altitude on cerebral and splanchnic oxygen saturation in critically ill children during air ambulance transport
Source: PLoS One. 2020 Sep 25;15(9):e0239272. doi: 10.1371/journal.pone.0239272 (PMC7518599; doi:10.1371/journal.pone.0239272)
Supplement: Attachment — Submitted filename: Response to Reviewers PONE.docx [file pone.0239272.s002.docx]

## Response to Reviewers PONE-D-19-35463

Thank you to both reviewers for Your constructive and thoughtful comments and suggestions. We are grateful for the opportunity to revise our article and agree that the manuscript can be much improved by changes according to Your suggestions.

Below written in red, are our responses to Your suggestions and comments.

Reviewer #1: Thank you for your careful data collection and thoughtful paper. I have these suggestions and questions that might guide enhancement:

1. Knowing the barometric pressure in the cabin would permit calculation of ambient PiO2. If available, these data should be included for each patient instead of, or in addition to, altitude.

Reply from author: Thank you for your suggestion to include data on barometric pressure in addition to cabin altitude. We agree with the reviewer and have added this data to Table 1.

2. Each patient also had pulse oximetry. Since rSO2 is a function not only of blood flow/metabolism but also of arterial saturation, it would be critical to the interpretation of rSO2 data to know what happened not only to the regional saturations, but to the differences between SpO2 and rSO2 – the best noninvasive estimation regional a-v difference, which is inversely proportional to the locoregional DO2/VO2. The authors have recognized this limitation, but I think there are ways to address this even given the lack of high-density time-synchronous measures:

In table 1, was the min/max SpO2 during transport? If so, one could calculate changes in cerebral and abdominal a-v difference using the min SpO2 and min rSO2 vs max SpO2 and max rSO2. You have data of altitude, SpO2, and rSO2 for at least 3 patients. Are there others? Could you make the above calculations on a subgroup with the SpO2 data available? I think this would strengthen your speculations about possible patterns and mechanisms.

Reply from author: We appreciate this thoughtful comment. We agree with the reviewer that an estimation of the a-v difference would have added important information and increased our ability to interpret the physiologic mechanisms at work. Indeed, data on SpO_2_ were gathered for every patient from routine monitoring at each phase of the study protocol. We were not able however to recover continuous data from the monitor in the same way we did from the NIRS measurements. During our data examination we tried to make calculations on a-v difference and fractional tissue oxygen extraction (FTOE) but came to the conclusion that the precision of the results was far from adequate. The single SpO_2_ measurement could be paired with any number of rSO_2_ continuous measures and the variability of rSO_2_ was so great that the SpO_2_-rSO_2_ differences varied significantly and made interpretation impossible. To evaluate the precision of estimated fractional tissue oxygen extraction the mean and standard deviation (SD) for rSO_2_-C and rSO_2_-A respectively, was calculated, using values during 2 minutes, starting 1 minute before the time of the SpO_2_ measurement. SD for SpO_2_ was assumed to be 0 since the value given on the pulse oximetry monitor is an average value and the very low variability during the entire registered period. The coefficient of variation (100*SD/FTOE) was calculated including propagation of errors involving subtraction and division according to JE Parks <http://www.phys.utk.edu/labs/plproerr.pdf>. A statistically significant increase in the coefficient of variation (CV) with decreasing fractional tissue oxygen extraction was found, as shown in the included supplementary figures. The CV was of such a magnitude that calculations on fractional tissue oxygen extraction bear no interest and there is a risk that erroneous conclusions could be drawn.

We hope that this further explanation convinces the reviewer that the manuscript would not be served by trying to include such imprecise data, and that clearer interpretation of a-v differences was not possible.

3. The somatic-cerebral difference or ratio is one way to ‘control’ for changes in SpO2 in patients who have normal circulatory anatomy and therefore no regional variation in SpO2, but infants with cyanotic CHD may have significant preductal/post-ductal SpO2 differences. The potential for differences in arterial saturation across the aortic arch confounds interpretation of regional rSO2 changes

Reply from author: The reviewer is quite correct in this important observation and we agree that the lack of pre -and post-ductal registrations with SpO_2_ during the flight protocol is a weakness. We have now acknowledged this as a limitation in Discussion, and to further distinguish this group we have in addition marked the cyanotic CHD patients with an * in both table 1 and 2 to make cyanotic and non-cyanotic patients more identifiable for the reader.

4. The left-shifted fetal oxyhemoglobin dissociation curve should reduce the altitude effect on pulmonary post-capillary oxygen saturation. Therefore, knowing the ambient PiO2 would help predict the pulmonary PpvO2 and SpvO2.

Reply from author: Thank you for this suggestion, this comment is physiologically sound and as noted above we have added information on barometric pressure in addition to cabin altitude in Table 1. Most children were neonatal patients with a median age of 12 days. Consequently, a significant proportion of hemoglobin (Hb) in the blood would be fetal if the child had not been transfused with adult blood. In a study aimed to determine whether it is necessary to modify the coefficients used in NIRS calculations when fetal Hb is present it was shown that adult Hb coefficients can be applied to newborns, irrespective of the level of fetal Hb, without introducing a significant error.

https://doi.org/[10.1203/00006450-199307000-00004](https://doi.org/10.1203/00006450-199307000-00004)

A Hb measurement was not documented in 25% of patients at the time of transport, this could influence both rSO_2_‐C and rSO_2_‐A levels, but since each patient served as its own control, we think this effect is insignificant for the results.

5. Obviously, changes in both total atmospheric pressure and the fio2 affect the PiO2. Any data to separate these effects would be helpful in interpreting an individual patient’s response to ‘altitude’ alone. This could be addressed partly by doing additional calculations in a subgroup restricted to those who did not have changes in fiO2 on transport

Reply from author: Thank you for this valuable comment. We have added information on barometric pressure and PiO_2_ for all patients in Table 1. For patients who had changes in FiO_2_ during flight, the PiO_2_ value given is with FiO_2_ added/increased. We have also included a Figure (Figure 4) on the few patients who had changes on FiO_2_ during transport. As can be seen the changes in FiO_2_ were relatively few and generally brief such that we don’t think they would have impacted tremendously on the general results seen. We are somewhat unclear on what additional calculations the reviewer is curious about – are there other calculations besides the additional data for barometric pressure, more detailed FiO_2_ information and PiO_2_ that the reviewer would like to see?

6. Please elaborate on the method to classify a significant change. I think you used the observed variability in each patient to calculate median and IQR, but over what time frame was variability? Was the change calculated from baseline, or from in-ambulance before takeoff, or multiply at altitude(s)? an alternative approach to examine a systematic relationship between altitude and rSO2 would be by regression, since you seem to have multiple measures of both altitude and rSO2 during ascent and descent for most if not all patients.

Reply from author: Thank you for this comment and the possibility to respond. The timeframes that we used including median time and IQR at each timeframe are now included in the manuscript and they were:

1) Departure from hospital ward - loading into ambulance - in ambulance - take-off. Median time 53 minutes (43-71)
2) Cruising altitude at 5000 feet or greater; 15 minutes (29-43)
3) Landing – unloading - in ambulance - arrival at receiving hospital; 53 minutes (41-61).

We found no general relationship between relatively long air transports at altitudes of 5000 feet or greater and decreases in oxygen delivery, i.e. all patients did not react with a decrease in NIRS, most likely because the type of medical condition is of importance. Therefore, we decided not to examine the data with regression analysis as a statistical method.

7. Somatic (renal, abdominal/mesentery) blood flow is highly affected by sympathetic tone. Since air transport has serious effects on patient state overall, the changes in abdominal NIRS might be expected regardless of altitude.

Reply from author: We thank the reviewer for raising this important subject and again agree with the physiologic rationale mentioned. If we understand the comment correctly one could imagine that general stress level could affect sympathetic tone, and more specifically events such as gastric feeding could affect abdominal blood flow. Feeding was generally done soon before the transport was initiated, and patients did not receive additional feeding during these relatively short transports. The general state of the patients was monitored during the protocol with TRIPS score for newborns (n=28) and Comfort-B scores for the other children and did not indicate a significant increase in agitation or stress for most patients, which speaks against other effects than altitude per see being the cause of the changes in abdominal NIRS.

8. The decision to void data at rSO2=15 or 95 is one way to deal with extreme values; the other is to leave them in. I understand that there are problems with either approach, but if a patient’s somatic rSO2 is progressively decreasing from 30’s->20’s->teens, I would not be inclined to disregard rSO2=15 as void. Likewise if cerebral rSO2 in increasing above 90, and lands pinned at 95%, I would generally interpret that as hyperemia. Exclusion of these extremes seemed to take out a significant part of the data.

Reply from author: We appreciate the comment and the opportunity to reply. During transport, floor effect was almost exclusively observed for rSO_2_-A. In this study we have not excluded rSO_2_-values >/= 95%. We excluded 5 patients from this study due to persistently showing values of rSO_2_-A=15% (See Figure 1). We have not excluded measurements if they were progressively decreasing from 30’s->20’s->teens.

9. One of the many challenges in transport, particularly air transport, would be reliability of measures. It might be helpful to present the number of minutes (or fraction of 6 second measures) during which data were not available (a value of 0 sent to the Invos data stream). It is my personal observation that NIRS measures are more reliably recovered than others during motion.

Reply from author: We thank the reviewer for addressing this important subject. We more closely studied the reliability of data in a previous methodological article (Journal of Clinical Monitoring and Computing (2018) 32:841–847 (<https://doi.org/10.1007/s10877-017-0094-z>).The median percentage of measurements = 0% was 2.1% (IQR 0.4–8.2%) for rSO_2_-C during flight and 4.4% (IQR 0.2–11.6%) during ground-ambulance transport. For rSO_2_-A, the median percentage of measurements = 0% during flight was 0.0% (IQR 0.0–1.1%) and during ground ambulance transport 0.5% (IQR 0.0–8.1%) respectively. Most of the patients in the methodological study are also included in the present study, therefore we did not address this subject in the current study but referred to the methodological study.

Reviewer #2: This article described a novel approach to noninvasive hemodynamic monitoring during neonatal and pediatric air ambulance transport. It has the potential to contribute to the field of neonatal/pediatric transport. The authors appropriately evaluated all patients as a whole as well as divided them into cyanotic and non-cyanotic patients, however it is suggested that they further define how they categorized patients into the cyanotic and non-cyanotic groups and what the mean/median pulse oximetry readings were for each group.

Reply from author: We thank the reviewer for this important comment and for the opportunity to reply. We categorized patients as cyanotic due to the presence of an intracardiac lesion or ductus arteriosus with a significant right to left shunt/mixing affecting the systemic saturation as measured by the SpO_2_. Diagnoses that were grouped into cyanotic CHD were: Transposition of the Great Arteries (TGA) n=3; Pulmonary Atresia n=2; Pulmonary Atresia & Tricuspid Atresia n=2; Total Anomalous Pulmonary Venous Return (TAPVR) n=1 and Truncus Arteriosis n=1. Accordingly, we have added this information into the manuscript as well as measurements of median pulse oximetry readings for both groups (the cyanotic and the non-cyanotic groups) before transport. To make cyanotic vs non-cyanotic patients more distinguishable to the reader, we have now marked the cyanotic CHD patients with an * in both table 1 and 2.

Additionally, there can be other changes to NIRS readings in patients not solely based on changes in altitude. These changes can be due to clinical severity. A transport risk index of physiologic stability (TRIPS) score could be used to further group patients based on their clinical severity and the effect on rSO2 during the transport.

Reply from author: We thank the reviewer for pointing out this important possible source of confounding. We have added information on TRIPS score for neonatal patients ≤ 30 days (n=28) at the time of transfer before flight, at ≥ 5000 feet and after flight in a table as well as in the text. For older children (n=11) PIM-score could have been appropriate, but unfortunately, we lack information on this. Older patients were monitored in the protocol with Comfort-B scores for level of sedation and necessary clinical interventions were equivalent with those for the newborn group.

The authors report statistically significant differences in both rSO2-C and rSO2-A when fiO2 was increased. It does not state in what direction these differences were appreciated. A figure would be helpful to illustrate the difference seen with increase in fiO2. Additionally, although the pulse oximetry information was not continuous data, it does seem that it correlated to the changes seen in rSO2-C and rSO2-A (figure 4). How would the addition of monitoring rSO2 add clinical utility over simply monitoring spO2? Equipment needs to be limited on transport due to space, weight and expense. The argument should be made for what NIRS would add to the clinical management of patients beyond our current monitoring system.

Reply from author: We thank the reviewer for this thoughtful and constructive comment and question. As requested by the reviewer, we have added a figure on those patients who needed extra oxygen during flight. We agree with the reviewer that it is important to more clearly address in what way and for whom NIRS would add to the clinical management during transport. In the revision we have tried to be more specific about this in the Discussion. Our opinion is that NIRS should be added, during transport, to those patients where the clinical management during anesthesia and intensive care would have included monitoring with NIRS, where measurements of venous saturations provide a means of titrating medications such as inotropes, vasoactive medicines and volume, or other similar situations of clinical significance. To other patients, NIRS monitoring during transport is probably superfluous and would not add any clinical utility.
