# Supplementary material for: Influence of altitude on cerebral and splanchnic oxygen saturation in critically ill children during air ambulance transport
Source: PLoS One. 2020 Sep 25;15(9):e0239272. doi: 10.1371/journal.pone.0239272 (PMC7518599; doi:10.1371/journal.pone.0239272)
Supplement: Attachment — Submitted filename: Response to Reviewers and Academic Editor PONE 200830.docx [file pone.0239272.s003.docx]

Response to Reviewers and Academic Editor PONE-D-19-35463

Thank you for Your constructive and thoughtful comments and suggestions. We are grateful for the opportunity to revise our article and agree that the manuscript can be much improved by changes according to your suggestions.

Below written in red, are our responses to Your suggestions and comments.

Reviewer #1: Thank you for your extensive and thoughtful revisions. Your key message, the change in cerebral and somatic rso2 at altitude, is somewhat obscured by the detail of data presented. Please consider a simple table that summarizes the median values of table 2 for all patients in the 3 conditions and consider a simple or interaction model to show the effect of cyanotic heart disease.

Reply from author: Thank you for your thoughtful suggestion to add a table that summarizes the median values listed in Table 2 for all patients. We agree with the reviewer that such information would help to summarize the data contained in the table and have followed the suggestion from the academic editor and added the median (IQR) for those with and without cyanotic heart disease at the bottom of Table 2.
